# Predicting prognosis using molecular profiling in estrogen receptor-positive breast cancer treated with tamoxifen

**DOI:** 10.1186/1471-2164-9-239

**Published:** 2008-05-22

**Authors:** Sherene Loi, Benjamin Haibe-Kains, Christine Desmedt, Pratyaksha Wirapati, Françoise Lallemand, Andrew M Tutt, Cheryl Gillet, Paul Ellis, Kenneth Ryder, James F Reid, Maria G Daidone, Marco A Pierotti, Els MJJ Berns, Maurice PHM Jansen, John A Foekens, Mauro Delorenzi, Gianluca Bontempi, Martine J Piccart, Christos Sotiriou

**Affiliations:** 1 Functional Genomics Unit, Jules Bordet Institute, Brussels, Belgium; 2 Peter MacCallum Cancer Center, East Melbourne, Victoria, Australia; 3 Machine Learning Group, Université Libre de Bruxelles, Brussels, Belgium; 4 NCCR Molecular Oncology, Swiss Institute of Cancer Research and Swiss Institute of Bioinformatics, Epalinges, Switzerland; 5 Guys Hospital, London, UK; 6 Molecular Cancer Genetics, Fondazione Istituto FIRC di Oncologia Molecolare (IFOM), Milan, Italy; 7 Erasmus MC-Daniel-JNI, Rotterdam, The Netherlands; 8 Department of Experimental Oncology, Fondazione IRCCS Istituto Nazionale dei Tumori, Milan, Italy

## Abstract

**Background:**

Estrogen receptor positive (ER+) breast cancers (BC) are heterogeneous with regard to their clinical behavior and response to therapies. The ER is currently the best predictor of response to the anti-estrogen agent tamoxifen, yet up to 30–40% of ER+BC will relapse despite tamoxifen treatment. New prognostic biomarkers and further biological understanding of tamoxifen resistance are required. We used gene expression profiling to develop an outcome-based predictor using a training set of 255 ER+ BC samples from women treated with adjuvant tamoxifen monotherapy. We used clusters of highly correlated genes to develop our predictor to facilitate both signature stability and biological interpretation. Independent validation was performed using 362 tamoxifen-treated ER+ BC samples obtained from multiple institutions and treated with tamoxifen only in the adjuvant and metastatic settings.

**Results:**

We developed a gene classifier consisting of 181 genes belonging to 13 biological clusters. In the independent set of adjuvantly-treated samples, it was able to define two distinct prognostic groups (HR 2.01 95%CI: 1.29–3.13; p = 0.002). Six of the 13 gene clusters represented pathways involved in cell cycle and proliferation. In 112 metastatic breast cancer patients treated with tamoxifen, one of the classifier components suggesting a cellular inflammatory mechanism was significantly predictive of response.

**Conclusion:**

We have developed a gene classifier that can predict clinical outcome in tamoxifen-treated ER+ BC patients. Whilst our study emphasizes the important role of proliferation genes in prognosis, our approach proposes other genes and pathways that may elucidate further mechanisms that influence clinical outcome and prediction of response to tamoxifen.

## Background

Breast cancers are biologically heterogeneous with regards to their clinical behavior and response to therapies. However, treatment-decision making for women diagnosed with breast cancer is still reliant on classical histopathological appearance and immunohistochemical markers that give little insight into tumor biology and potential response to treatment. There are a few biomarkers routinely used that can predict response to commonly prescribed therapies. The presence of estrogen receptors is the best indicator of response to anti-estrogen agents such as tamoxifen. However, 30–40% of women with estrogen receptor-positive breast cancer (ER+BC) will develop distant metastases and die despite tamoxifen treatment. The underlying biological mechanisms of resistance to tamoxifen are incompletely understood.

Gene expression profiling of tumors appears to be a promising new strategy for predicting clinical outcome in breast cancer patients. Recent studies have proposed that the heterogeneity of clinical response can be correlated with different molecular "portraits" [1,2]. Gene signatures have been developed that can distinguish subgroups of patients with different prognoses or response to chemotoxic and antiestrogen agents. However, issues have emerged since these initial studies relating to design and validation of gene classifiers [3], particularly the small numbers of patient samples used to derive the classifier and the little overlap in these gene signatures. Furthermore, it has been shown that membership in a prognostic gene list is not necessarily indicative of a gene's importance in cancer pathology [4]. Extracting biological meaning from whole genome molecular profiling remains a significant challenge.

We have recently shown that in ER+ BC, its proliferative status is the most important predictor of prognosis in these women [5]: highly proliferative tumors have a worst clinical outcome, either with or without systemic treatment. However, proliferation is a downstream consequence and the understanding of the upstream activators is essential for advancing biological knowledge and development of targeted approaches that may be tested in the clinical setting, potentially in combination with anti-estrogen agents. In this study, we hypothesized that developing a gene classifier using clusters of correlated genes as single variables may allow for both prediction of clinical outcome in tamoxifen-treated patients and facilitate new biological understanding of resistance mechanisms as these clusters could represent biological networks or pathways. Furthermore, we assessed the performance of our classifier on several independent data sets of tamoxifen-treated samples, both in the adjuvant as well as advanced setting. These were obtained from a number of institutions and samples had been hybridized on varying microarray platforms.

## Methods

### Tamoxifen-treated dataset used in development of the classifier

The dataset used for training the classifier consisted of 255 early-stage (stage I, II) BC samples, diagnosed between 1980 and 1995, all of whom had received tamoxifen only as their adjuvant treatment (hereby referred to as the "tamoxifen-treated dataset"). The demographics can be found in [Additional file 1], and data processing methods are described in Loi et al. [6] as a large proportion of this dataset has been previously used in another research study. The raw data for the tamoxifen-treated dataset are available at the GEO database (accession number GSE6532). This dataset contained samples from the John Radcliffe Hospital (OXFT), Oxford, United Kingdom, Guys Hospital (GUYT), London, United Kingdom and Uppsala University Hospital (KIT), Uppsala, Sweden. All samples had been hybridized using Affymetrix U133 Genechips™ (HG-U133A, B for OXFT and KIT, and PLUS2 for GUYT). All samples were required to be estrogen (ER) and/or progesterone receptor (PR) positive by ligand-binding assay and had been prescribed tamoxifen monotherapy for 5 years post diagnosis as adjuvant therapy. The cut-off value for classification of patients as positive or negative for ER and PR was 10 fmol per mg protein. The primary endpoint used for generating the classifier was the first distant metastatic event (distant metastasis free survival, DMFS), as survival can be confounded by local recurrence and treatments given at relapse. Each hospital's institutional ethics board approved the use of the tissue material for the purposes of this research study.

### Statistical methods

**Figure 1:**
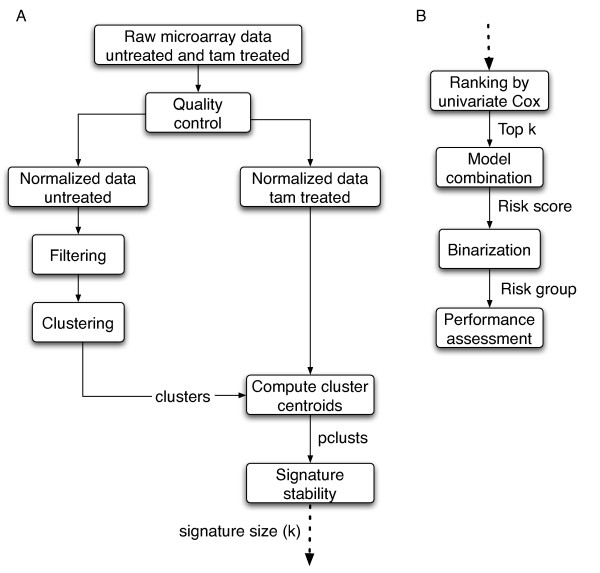
**Overview of the analysis design**. (a) First part of the analysis including quality controls, normalization, preliminary clustering performed on the untreated dataset, computation of the cluster centroids on the tamoxifen treated dataset, and estimation of signature stability with regards to signature size, using cross-validation. (b) Second part of the analysis including the classifier development, performance assessment by cross-validation and performance assessment on independent validation data sets.

Figure 1a and 1b summarize the method used to develop the gene classifier.

#### Preliminary clustering

We used a clustering method in order to identify clusters of highly correlated genes, prior to feature selection and model building as we hypothesized that this would to reduce the number of variables, increase signature stability, allow platform independency and to preserve biological interpretation [8]. Preliminary clustering was performed on separate dataset consisting of 137 samples from untreated women with early stage breast cancer (data available from GEO database, accession number GSE6532). These samples were not used in the signature development to avoid any possible overfitting when performing the cluster identification. Control probe sets and those absent in at least 95% of the samples were removed. The data set was then filtered based on overall variance with the top 20% of probe sets selected for further clustering. Hierarchical clustering with Pearson correlation similarity metric and complete linkage was used. The generated dendrogram was then cut at a height of 0.5. Clusters were discarded if there were less than 5 known genes (as per Unigene) per cluster. After this procedure, a total of 110 clusters were obtained for signature development [Additional file 2a]. Of note, these clusters were able to be reproduced in the tamoxifen-treated population (data not shown). The cluster centroid, i.e. the average expression level of all the probes per cluster, was then obtained for each cluster in the tamoxifen-treated dataset. Each cluster was subsequently treated as a single variable called a "probe cluster" (*pclust*).

#### Feature selection

Although the preliminary clustering significantly reduced the dimensionality of the data, the number of features remained too large to efficiently build the classifier. The selection of the most relevant pclusts was performed using a ranking based on the likelihood ratio statistic of univariate Cox model. The Cox model specifies the hazard of a patient *i *as

*λ*_*i *_(*t*) = *λ*_0_(*t*)exp(*β pclust*_*j*_),

where *λ*_0_(*t*) is the baseline hazard assumed to be equal for all patients (proportional hazards), and *pclust*_*j *_is the *j*th pclust of patient *i*. The likelihood ratio statistic is twice the difference in the log partial likelihood between the null model (*β *is equal to 0) and the model with estimate of *β*. The only parameter of this feature selection is the signature size, i.e. the number *k *of pclusts that will be used to build the classification model.

#### Signature size and stability

Signature size was set in order to maximize a stability criterion using a multiple 10-fold cross-validation algorithm (M10FOLDCV) on the tamoxifen-treated dataset after randomization of the order of patients in the data set. For each signature size, a criterion designed to estimate the stability of a signature was computed, as recently introduced by Davis and colleagues [9]. For a given signature size *k*, let *P *be the list of all pclusts. Let *freq(p) *be the number of sampling steps in which the probe cluster *p *∈ *P *has been selected out of a total of *m *sampling steps. The set *P *is sorted by frequency into the set *p*_(1)_, *p*_(2)_,..., *p*_(*n*) _such that *freq*(*p*_(*i*)_) ≥ *freq*(*p*_(*j*)_) if *i *<*j *where *i*, *j *∈ {1, 2,..., *n*}. The stability statistic for a signature size *k *is defined as

$$Stab(k)=\frac{\sum_{i=1}^{k}freq(p_{i})}{k\;m}$$

The *Stab *statistic is equal to 1 if the same signature is always selected over M10FOLDCV given a signature size and $\frac{1}{m}$ if there is no overlap. It must be noted that *Stab *statistic converges to 1 as the signature size converges to the total number of variables. Therefore, *k *was chosen as a trade-off between signature size and stability, i.e. a signature size exhibiting maximal possible stability and being smaller than the total number of variables.

#### Model building

As multivariate survival models using microarray data are prone to overfitting, we built the model by combining the univariate Cox models computed during feature selection [8]. Each univariate model is defined as *β pclust*_*j*_, also referred as *risk score *in the literature. We used the sum rule as this method outperforms more complex combination schemes [7]. We set all the weights to 1 and computed the combined risk score as $\sum_{j=1}^{k}\beta pclust_{j}$.

#### Method evaluation

To avoid over-optimistic estimation of prediction accuracy, a leave-one-out cross-validation (LOOCV) and M10FLODCV procedures were used. As LOOCV does not depend on the order of patients in the dataset, these results will be discussed in details.

### Independent validation data sets

Four independent validation sets were used to assess the performance of the classifier. These demographics are shown in [Additional file 3].

#### Guy's hospital dataset (GUYT2)

This external validation set was kindly provided by the Guy's Hospital, London, United Kingdom, consisting of 77 patients diagnosed with early stage breast cancer and treated with adjuvant tamoxifen monotherapy. Samples were hybridized using Affymetrix U133PLUS2 Genechips™ according to standard Affymetrix protocols. Gene expression values from the CEL were normalized by use of the standard quantile normalization method in RMA [10] and are available from GEO database, accession number GSE9195.

#### Dataset of Ma et al. (Ma)

This dataset consisted of 60 patients diagnosed at the Massachusetts General Hospital, Boston, United States of America, and who were treated with adjuvant tamoxifen monotherapy. The samples were hybridized on the Agilent microarray platform and have been previously described [11]. The raw data was obtained at the GEO database (accession number GSE1378).

#### Dataset of Reid et al. (Reid)

This external validation set was kindly provided by the Department of Experimental Oncology, Istituto Nazionale per lo Studio e la Cura dei Tumori, Milan, Italy, consisting of 113 patients who had received adjuvant tamoxifen monotherapy. Samples were hybridized on their local cDNA microarray platform. Part of this dataset has previously been published [12]. We were unaware of the clinical data at all times and the survival analyses were performed in Milan.

#### Dataset of Jansen et al. (Jansen)

This dataset consisted of 112 patients diagnosed at the Erasmus MC, Rotterdam, Netherlands and who were treated with tamoxifen in the metastatic setting as first line hormonal therapy. Tumour response previously described [13] included both complete and partial responses and progressive disease. Samples were hybridized on 18 K human cDNA arrays manufactured at the Central Microarray Facility at the Netherlands Cancer Institute, Rotterdam, Netherlands. The raw data was kindly supplied by our Rotterdam colleagues.

Mapping across microarray platforms was done using the "Cleanex" database [14] to retrieve corresponding gene symbols and Affymetrix probe sets.

### Statistical analysis

Although the risk score can be used as a continuous variable, we divided the dataset into two prognostic groups to generate a high or low risk status as this allowed us to estimate hazard ratios and produce Kaplan Meier curves. For the purposes of this study, a binary classification was generated using a 70:30 cutoff that is, 70% of samples would be considered low risk (hence, the majority of these patients would still suitable for tamoxifen) and 30% high risk of relapse on adjuvant tamoxifen monotherapy. This cutoff was an arbitrary figure chosen by the authors to balance the cost of tamoxifen vs. other more expensive endocrine agents against relapse risk. The results shown in this manuscript were from analyses using the 70:30 cutoff for tamoxifen-treated and the external validation GUYT2 datasets, though similar results were obtained with a 50:50 cutoff. However, the samples in the Ma and Reid datasets were chosen to be balanced for recurrences within 5 years and non-recurrences after 6 years (case control studies, a non-consecutive series). Therefore, a 50:50 cutoff was used to take into account for the balanced number of events. Performance of the classifier computed by LOOCV was assessed using Kaplan-Meier survival curves and log rank p-values. The overall performance of the classifier in the three adjuvant data sets was estimated using classical meta-analysis methods [15].

Hazard ratios (HRs) for the risk groups defined by the classifier were calculated using a Cox's regression stratified by clinical center to account for possible heterogeneity in patient selection or other potential confounders among the various centers. For each independent validation data set, the HR (with their 95% confidence intervals [CI]) was displayed on a forest plot and tested for heterogeneity using a chi-square test [15]. HRs were then combined using the inverse variance-weighted method with fixed effect model [15] to compute an overall HR.

To establish if the model predicted response to treatment, a univariate logistic regression model was used with the risk score as explanatory variable. Significance was determined by the Wald test and a false discovery rate (FDR) < 0.05.

Statistical analysis was performed using SPSS statistical software package version 13.0 and the R software package version 2.3 [16].

### Correlation with the grade gene expression index (GGI)

The Spearman's correlation between the risk scores produced by the predictor and that produced by GGI, previously described in Sotiriou et al, 2006 [5], was calculated to assess the contribution of proliferation-related genes to the prognostic ability of the current predictor.

### Network and pathway analysis

Analysis of gene interactions for each cluster of the final classifier was performed using Ingenuity Pathways Analysis (IPA) tools version 3.0 [17]. Affymetrix probe sets of each cluster were used as input to generate biological networks based on a curated list of molecular interactions in IPA. IPA then calculated a significance value for enrichment of the functional classes and canonical pathways generated for each of these networks. Only significant functions and pathways are shown.

## Results

### Predictor development

**Figure 2:**
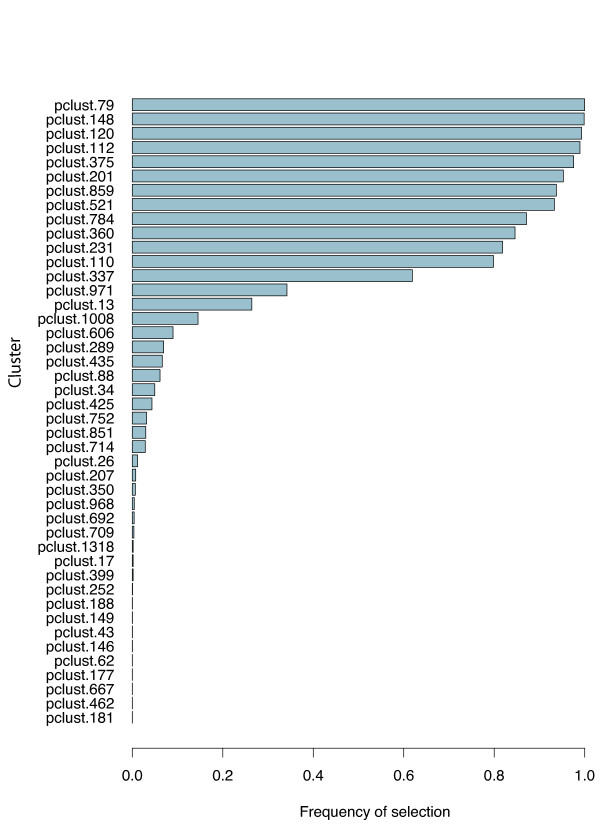
**Signature stability**. Signature stability demonstrating frequency of selection for the various clusters in multiple 10-fold cross-validations.

Gene expression profiles of the 255 patients in the training set were used to derive the predictor. A signature of 13 clusters was assessed to be highly stable [Additional file 4] and hence chosen for further predictor development. In terms of performance, the best predictor used 45 clusters but a signature size of 13 performed similarly and had the advantage of using fewer clusters [Additional file 51]. Figure 2 shows the frequency of selection of each cluster in the M10FOLDCV process. All of the 13 clusters incorporated in the final predictor were the most frequently selected during the training phase.

### Classifier performance on the training set

**Figure 3:**
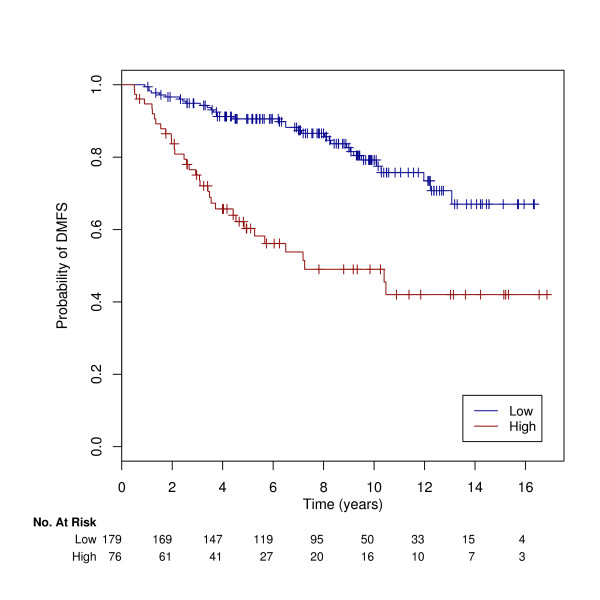
**Survival curves for training set**. Kaplan Meier curves for the binary classification computed using leave-one-out cross-validation on the tamoxifen-treated dataset (n = 255). The two survival curves were significantly different according to the log rank test (p < 0.0001).

A risk score derived from 13 clusters was developed to predict the patient's risk of developing distant metastases as high or low risk. The final model consisted of 13 clusters and 239 associated probe sets (181 genes, [Additional file 2b]). The hazard ratio [15] for the occurrence of distant metastases using LOOCV was 3.86 (95% CI: 2.32–6.41) p < 0.0001 using the classifier as a binary variable. Results using M10FOLDCV were slighter lower however the difference was small (HR: 3.28, 95%CI: 2.66–3.84, p < 0.0001). We further assessed the performance of our method by dividing the dataset into separate populations according to institution. The performance of the classifier algorithm on each population and corresponding 3-year DMFS is shown in Table 1, providing supporting evidence that the classifier algorithm is not population dependent. The survival curves estimated by Kaplan-Meier analysis are shown in Figure 3.

**Table 1:** Performance of the classifier. Performance of the 13 clusters classifier algorithm re-training and validation on the separate institutional populations using both leave-one-out and multiple 10-fold cross-validations.

**Training set (total/events)**	**Validation set (total/events)**	**Hazard ratio (95%CI)**	**Log rank p value**	**Distant Metastases Free Survival for low risk group only#**		
				
				DMFS at 3 years	DMFS at 5 years	DMFS at 10 years
OXFT (99/19)	KIT/GUYT(156/48)	2.17 (1.2–3.91)	<0.00001	91%	87%	79%
KIT (69/20)	OXFT/GUYT (186/47)	4.07 (2.23–7.41)	<0.00001	96%	92%	88%
GUYT (87/28)	OXFT/KIT (168/39)	5.93 (3.0–11.75)	<0.00001	93%	89%	82%
KIT/GUYT (156/48)	OXFT (99/19)	14.59 (5.38–39.5)	<0.00001	97%	94%	91%
OXFT/GUYT (186/47)	KIT (69/20)	3.44 (1.36–8.67)	0.005	96%	92%	84%
OXFT/KIT(168/39)	GUYT (87/28)	2.23 (1.05–4.71)	0.03	96%	92%	84%
Leave-one-out cross validation (255/67)*		3.86 (2.32–6.41)	<0.0001	94%	91%	84%
**Multiple 10-fold cross-validation (255/67)**		3.23 (2.66–3.84)	<0.0001	94%	90%	83%

# as estimated by Kaplan Meier survival curves.

Patients samples obtained from: OXFT: John Radcliffe Hospital, Oxford, UK; KIT Uppsala University hospital, Uppsala, Sweden; GUYT Guys hospital, London, UK.

• Reported results.

Univariate Cox regression analysis of the training set with the classifier and clinico-pathological prognostic factors known are shown in Table 2. The classifier gives the strongest HR with histological grade (HR = 1.77, 95%CI: 1.17–2.68, p = 0.007) and tumor size (HR = 2.18, 95%CI: 1.27–3,75, p = 0.003) also significant. In the multivariate model, the classifier retained its significance as an independent variable for prediction of distant recurrence (HR = 3.26, 95%CI: 1.76–6.05, p = 0.0002).

**Table 2:** Cox regression analysis. Univariate and multivariate Cox regression analysis for time to distant metastases in 255 patients.

	**Univariate analysis**		**Multivariate analysis#**	
		
	Hazard ratio (95%CI)	p	Hazard ratio (95%CI)	p
Histological grade (1 vs. 2 vs. 3)	3.14 (1.37–7.17)	0.007	0.94 (0.36–2.42)	0.9
Tumor size (≤ 20 mm vs. ≥ 20 mm)	2.18 (1.27–3.75)	0.005	1.58(0.83–3)	0.2
Nodal status (positive vs. negative)	1.62 (0.95–2.79)	0.08	1.30 (0.71–2.37)	0.4
ER high vs. low expression	0.86 (1.18–0.522)	0.5	0.97 (0.56–1.7)	0.9
PgR high vs. low expression	0.42 (0.25–0.7)	0.0007	0.49 (0.26–0.9)	0.02
HER2 high vs. low expression	0.88 (0.55–1.42)	0.6	0.66 (0.37–1.18)	0.2
13 cluster gene classifier*	3.86 (2.32–6.41)	<0.0001	3.26 (1.76–6.05)	0.0002

#Multivariate model contained included 210 patients due to missing values, stratified by population.

*Binary classification using leave-one-out cross-validation.

**Age was not included in the model as 92% of patients were ≥ 50 years of age.

ER: estrogen receptor status represented by ESR1 Affymetrix probe set 205225_at.

PgR: progesterone receptor status represented by PGR Affymetrix probe set 208305_at.

HER2: represented by ERBB2 Affymetrix probe set 216836_s_at.

For ER, PgR and HER2, high vs. low expression groups was defined by generating groups at the median value.

### Independent validation on external datasets: a meta-analysis

**Figure 4:**
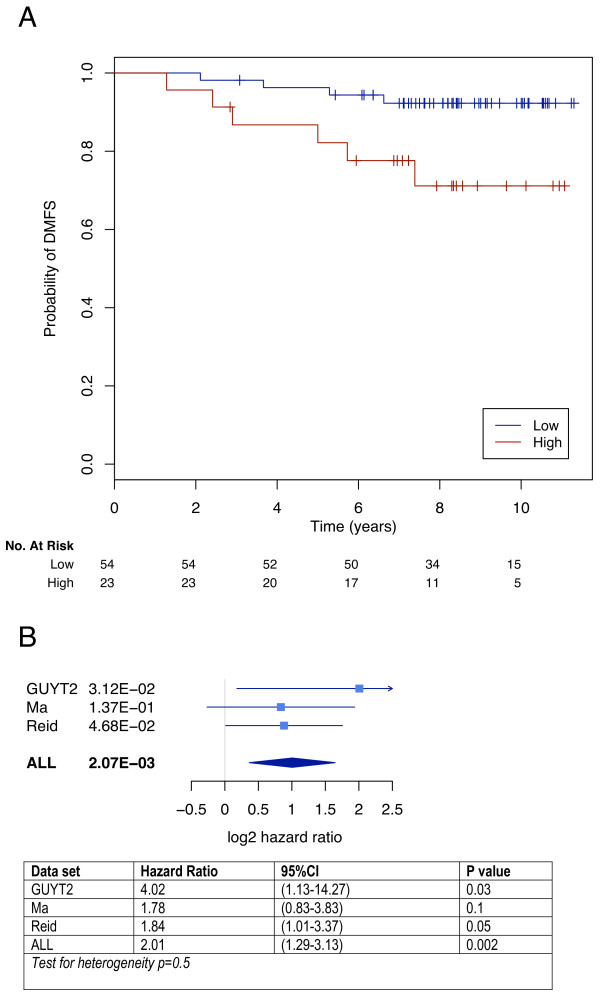
**External validation of the classifier**. (a) Kaplan Meier curves for the GUYT2 dataset. The two survival curves were significantly different according to the log rank test (p = 0.03). (b) Forest plots of hazard ratios obtained from the three independent validation datasets.

Validation of the classifier was performed on three independent data sets (GUYT2, Ma, and Reid) consisting of 250 samples taken from women at diagnosis and who had received the same adjuvant systemic therapy. These three datasets had been hybridized using different microarray platforms. The number of probe sets that could be mapped per cluster is shown in Table 3. Whilst the GUYT2 dataset was obtained from a consecutive series of patients (results by Kaplan Meier analysis shown in Figure 4a), the samples in the Ma and Reid datasets were chosen to be balanced for recurrences within 5 years and non-recurrences after 6 years (case control studies, results from Kaplan-Meier analysis shown in [Additional file 6]. The overall performance of the classifier in the 3 datasets is shown in Figure 4b. In the 250 women treated with adjuvant tamoxifen, the classifier was able to define two distinct prognostic groups (HR 2.01, 95%CI: 1.29–3.13, p = 0.002). Interestingly, in the Affymetrix validation dataset (GUYT2), where all probe sets could be mapped, the performance of the classifier was the highest (Figure 4a: HR 4.02, 95%CI: 1.13–14.27, p = 0.03), suggesting that the validation may have been limited by technical factors. A multivariate analysis comparing other prognostic factors with the gene classifier was not performed due to a large number of missing values. However overall, this meta-analysis still provides evidence that our classifier has significant clinical value for prediction of distant relapses in patients treated with adjuvant tamoxifen monotherapy.

**Table 3:** Mapping. Number of probe sets able to be mapped across datasets during independent validations.

**Cluster no.**	**Total probe sets present in classifier**	**Mapped**			
		
		**GUYT2 dataset**	**Ma dataset**	**Reid dataset**	**Jansen dataset**
79	7	7	3	1	2
148	45	45	23	23	15
112	14	14	5	4	9
120	38	38	18	15	16
375	8	8	4	3	3
201	17	17	5	8	6
521	19	19	10	8	5
784	7	7	1	1	1
859	7	7	4	2	2
360	14	14	4	2	0
231	26	26	8	4	9
110	30	30	13	3	10
337	7	7	4	1	1

**Total n (%)**	239 (100%)	239 (100%)	102 (42.6%)	75 (31.4%)	73 (30.5%)

### Prediction of response in metastatic breast cancer patients treated with tamoxifen

In order to delineate whether our classifier was predicting response to tamoxifen and/or the intrinsic aggressiveness of a breast tumor (prognostic), we applied our classifier to a data set of women who had received tamoxifen in the advanced setting where response to the treatment was clearly defined [13]. Twenty-nine of the 79 probes that could be mapped were significantly associated with clinical response (complete or partial vs. progressive disease, false discovery rate (FDR) < 0.05) and 4 cluster groups seemed to have some predictive ability (FDR < 0.15, *pclusts *148,120,375,201). However, overall, we found that our classifier had no discrimination ability in this group of patients. Interestingly one cluster centroid, cluster 375, was significantly associated with response (FDR = 0.008), suggesting that this cluster of 3 genes [see Additional file 2b] could predict response to tamoxifen treatment. These results could imply that our classifier is mainly prognostic, though as only 30.5% of probe sets were able to be mapped from the cDNA platform, technical limitations could have significantly contributed to these results.

### Correlation with the grade gene expression index (GGI)

The GGI is an algorithm which can quantify the expression of proliferation genes in a breast tumor [5]. Given that many current prognostic predictors derive a significant proportion of their discriminatory ability from proliferation-related genes [5], we were interested to assess this in our current predictor. Despite the different discovery methods, the groupings produced by our classifier and the GGI were highly correlated: (GUYT2 0.91; Reid 0.86; Ma 0.69; Jansen 0.55; all p-values < 0.05), suggesting a significant proportion of its predictive power can be attributed to cell cycle-related genes.

### Functional analysis

The biological functions of each of the 13 clusters were analyzed in the context of a curated list of published molecular interactions by IPA. Table 4 lists the high level functions and associated canonical pathways with statistically significant enrichment for each cluster. As seen, there are a number of gene clusters related to cell cycle function, supporting our finding above. Cluster 110 contains genes that have previously been associated with chemotaxis and invasion of breast cancer cell lines (SLIT2, RECK) [18,19], as well as genes related to the extracellular matrix (ECM2, COL4A1). Less well characterized is the role of lipid metabolism (cluster 79) and immunological aspects in the differential response to tamoxifen (clusters 784, 375) though TNF alpha and TGF beta have previously been implicated in breast cancer development and progression [20]. Cluster 375, though small, is of interest, given its performance in the Jansen dataset. Functional analysis suggests that these genes (TGFBR4, PTGER4, C3, GNG2) are mainly involved in cellular inflammatory response and could be particularly important in determining the host's response to tamoxifen. The presence of gene cluster in our predictor that allude to other biological pathways apart from cell cycle function may facilitate further understanding of the upstream mechanisms behind tamoxifen resistance.

**Table 4:** Functional analysis. Functional analysis of the 13 clusters from the gene signature (for full gene list [see Additional file 2b]).

**Cluster no.**	**Top Network overall**	**Top high level function**	**Top canonical pathway**	Number of focus genes able to be mapped*
79	Cancer Inflammatory disease Cell cycle	Lipid metabolism Molecular transport	cAMP mediated signaling	3
148	Cancer Immune response	Cell cycle	1 carbon pool by folate	28
112	Gene expression	Gene expression Protein synthesis	EGF signaling	10
120	Cell cycle Cellular movement	Cell cycle	G2/M checkpoint	28
375	Cellular movement, inflammatory disease	Carbohydrate metabolism	TGF-beta signaling	4
201	DNA recombination and repair	Cell cycle	G1/S checkpoint	11
521	Cell cycle	Cell cycle	G1/S checkpoint	14
784	Cell death	Cell morphology Cellular development	IL4 signaling	3
859	Gene expression	Cell morphology	None given	4
360	DNA recombination and repair	Cell cycle	None given	6
231	Cell to cell signaling and interaction	Embryonic development	IGF1 pathway	13
110	Cell death Cellular development	Cancer Inflammation	PDGF signaling	14
337	Cell morphology	Cellular function and maintenance	None given	1

* note that clusters (*pclust*) often contained probe sets that represented the same gene

## Discussion

Developing gene signatures that are stable, are effective at distinguishing prognostic groups and provide important biological information from whole genome microarray data remains a significant challenge. We propose a method which has similarities to a technique proposed by Bair and colleagues [21,22], in combination with an estimation of signature stability [9] and to our knowledge, the largest dataset of ER+ patients homogenously treated in an attempt to address these issues. Whilst Bair et al. [21] used the clinical data to define a subset of survival-related genes prior to clustering, we performed an initial unsupervised clustering procedure to form the clusters which could act as biological networks, which were then used as single variables to build the classifier. We hypothesized that this would limit the effect the training set has over the final selection of genes for inclusion in the classifier [4] and allow a larger gene list for biological hypothesis generating. The inclusion of an assessment of "stability" facilitates determination of the most robust variables and hence presumably important biological information.

With this method, we were able to develop and validate a gene classifier that could predict which patients with ER+ BC were at high risk of relapse despite tamoxifen treatment. Importantly, we were able to validate the classifier on independent samples utilizing raw data from different microarray platforms using a meta-analytical approach. Demonstration of prognostic ability is important if we are to assemble gene lists from microarray data for biological hypotheses generation and potential laboratory experimental validation, which was one of the most important aims of this study. Validation of gene classifiers with independent samples from which they were developed from is a major challenge for microarray studies, especially those with clinical implications, and combining multiple datasets can be difficult due to different patient populations, sample preparation and microarray platforms. Our study uses one of the largest training and validation sets reported in the literature on tamoxifen (only) treated patients.

Whilst, in the future we may have a microarray-based diagnostic test incorporating all 181 genes in the 13 clusters, at present the routine use of this technology is not logistically feasible. However, the advantage of our approach is that as each cluster consists of a group of genes that are highly correlated and hence effectively act as one covariate. Thus, a diagnostic test of just 13 genes (one per cluster) could be developed for clinical use if desired, even though for biological study the researcher would be more interested in all the genes per cluster. To demonstrate this, we took a series of 13 individual probe sets (one per cluster) and correlated their performance with the full classifier on the training set of 255 patients. The median correlation was 0.94 (range: 0.88–0.97). The top 26 ranked 13-gene classifiers (with a correlation ranging from 0.95–0.97) and their corresponding probe sets are listed in [Additional file 7]. These "simple" tests will require further independent assessment but could be validated using immunohistochemistry or quantitative RT-PCR and are attractive option for potential clinical implementation.

Due to the pressing clinical need, several other investigators have also developed gene predictors that can predict outcome in ER+ BC treated with adjuvant tamoxifen monotherapy [11,13,23,24]. These studies have used a variety of bioinformatics approaches to develop these gene signatures. These range from a candidate gene approach [24], selection of genes using a biological approach [23] and similar to our study, a discovery-based approach using supervised analyses correlated with clinical outcome [13]. Likewise, different patient populations were used in the development process. Ours is the only study to use a large, consecutive series of patients as a training set as opposed to samples obtained from a clinical trial [24]; or a case control population [11]. Only one of these reported gene classifiers has undergone noteworthy clinical validation [24], however unfortunately these genes provide no new potential therapeutic targets or insights into the underlying biology. Of note, we have previously published that proliferation-related genes are the common biological thread linking many of these currently published classifiers [5,6]. Our current classifier also has a significant amount of cell cycle genes, and is highly correlated with the GGI, but one of the aims of this study was to identify other potential biological mechanisms upstream of proliferation. All the clusters in the final classifier were the most common chosen during the cross-validation process suggesting the presence of other strong biological signals. Further experimental validation in in-vitro and in-vivo models will be required to test these hypotheses and their relevance to the clinical question. Interestingly, the cluster 375 was significantly predictive in the dataset of metastatic breast cancer patients treated with tamoxifen as first line treatment for relapsed disease. However, we were not able to validate the full gene classifier. The best approach on distinguishing prognosis versus therapy prediction using gene expression profiling remains unclear. It is possible that developing a predictor of true response to therapy may only be possible using samples from a randomized trial in the metastatic setting where response can be clearly defined and transcriptional profiles can be compared with an untreated control group.

## Conclusion

Using a discovery-based whole genome approach, we have developed and validated a gene classifier that can distinguish patients at high risk of distant metastasis despite adjuvant tamoxifen monotherapy. In the future, these poor prognosis patients could be selected for prescription of other treatment modalities, such as chemotherapy and/or biological agents. In this study we propose an approach which has the advantage of facilitating both signature stability and biological interpretation. These are critical issues in the challenging task of building gene predictors for breast cancer patients as we endeavor to delineate meaningful biological and clinically useful information from the microarray-produced data.

## Authors' contributions

SL, BH–K was responsible for the design and execution of the study, collation of study materials, the microarray analysis of study samples, the collection, assembly and verification of the data, data and statistical analysis and interpretation and final manuscript writing; CD assisted with the collation of study materials, the microarray analysis of study samples, data analysis and interpretation and final manuscript writing; PW assisted with the microarray analysis of study samples, data analysis and interpretation; FL provided technical support with the microarray analysis; AMT, PE, CG, KR, supplied the tissue samples from Guy's Hospital; JR, MGD, MAP performed the bioinformatics analysis using the Reid dataset in Milan; EMJJB, MPHMJ, JAF provided the microarray raw data for the Jansen dataset, and assisted with the data analysis and interpretation; MJP provided the study funding; MD, GB, and CS supervised the study. CS conceived the idea for the study. All authors read and approved the final manuscript.

## Supplementary Material

Additional file 1 **Training dataset characteristics**. Summary of patient and tumor characteristics of the 255 patients used in the development of the classifier. Click here for file

Additional file 2 **Probe clusters**. (a) Full annotated gene list of all clusters (probe clusters; "*pclust*") used for classifier development after preliminary clustering and filtering on the separate dataset of 137 samples. (b) Full annotated gene list of probe clusters (*pclust*) used in final classifier and global model. Click here for file

Additional file 3 **Independent validation datasets characteristics**. Summary of patient and tumor characteristics of the four independent validation sets. Click here for file

Additional file 4 **Signature size stability**. Evolution of *Stab *criterion with respect to the signature size using multiple 10-fold cross-validation. The vertical dashed line represents the stability of the ranking using 13 *pclusts*. Click here for file

Additional file 5 **Signature size performance**. Evolution of the log2 hazard ratio with respect to the signature size using multiple 10-fold cross-validation. The vertical dashed line represents the performance of the classifier using 13 *pclusts*. The horizontal red dashed line represents the limit of statistical significance. Click here for file

Additional file 6 **External validation of the classifier (Ma, Reid and Jansen datasets)**. (a) Kaplan Meier curves for Ma et al. The risk group was defined by the classifier using a 50:50 cutoff. The two survival curves were not significantly different according to the logrank test (p-value of 0.1). (b) Kaplan Meier curves for Reid et al. The risk group was defined by the classifier using a 50:50 cutoff. The two survival curves were significantly different according to the logrank test (p-value of 0.05). (c) Kaplan Meier curves for Jansen et al. The risk group was defined by the classifier using a 50:50 cutoff. The two survival curves were significantly different according to the logrank test (p-value of 0.25). Click here for file

Additional file 7 **Simplified classifiers**. Top correlated "simplified" classifiers of 13 genes (one probe set per cluster) and histogram displaying correlation between "simple" classifiers and final model. Click here for file
