# Supplementary figures and images for: Predicting prognosis using molecular profiling in estrogen receptor-positive breast cancer treated with tamoxifen
Source: BMC Genomics. 2008 May 22;9:239. doi: 10.1186/1471-2164-9-239 (PMC2423197; doi:10.1186/1471-2164-9-239)

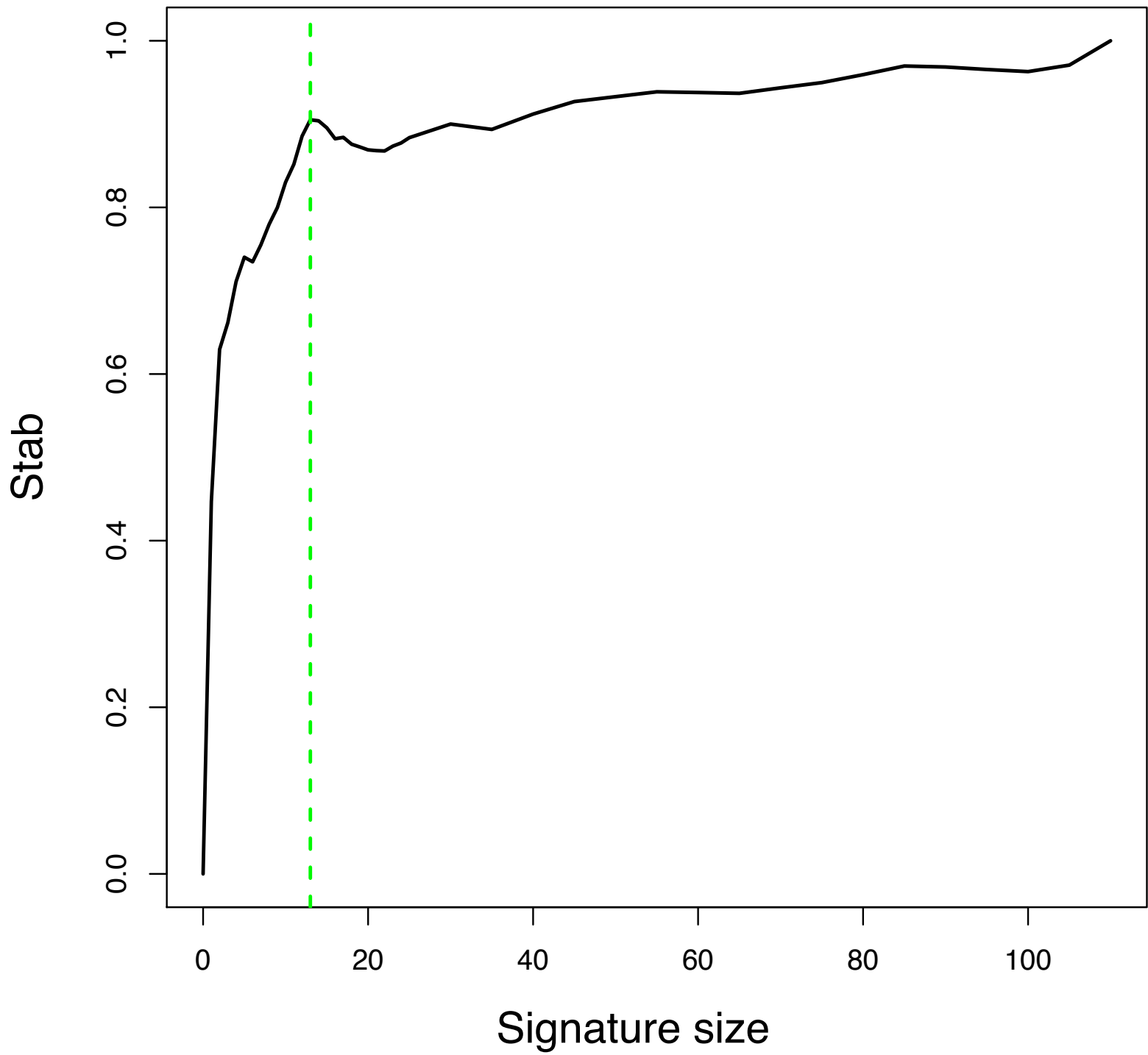

Supplement: Additional file 4 — Signature size stability. Evolution of Stab criterion with respect to the signature size using multiple 10-fold cross-validation. The vertical dashed line represents the stability of the ranking using 13 pclusts. [file 1471-2164-9-239-S4.pdf]

# Hazard ratio (CI) wrt signature size 10FOLD CV

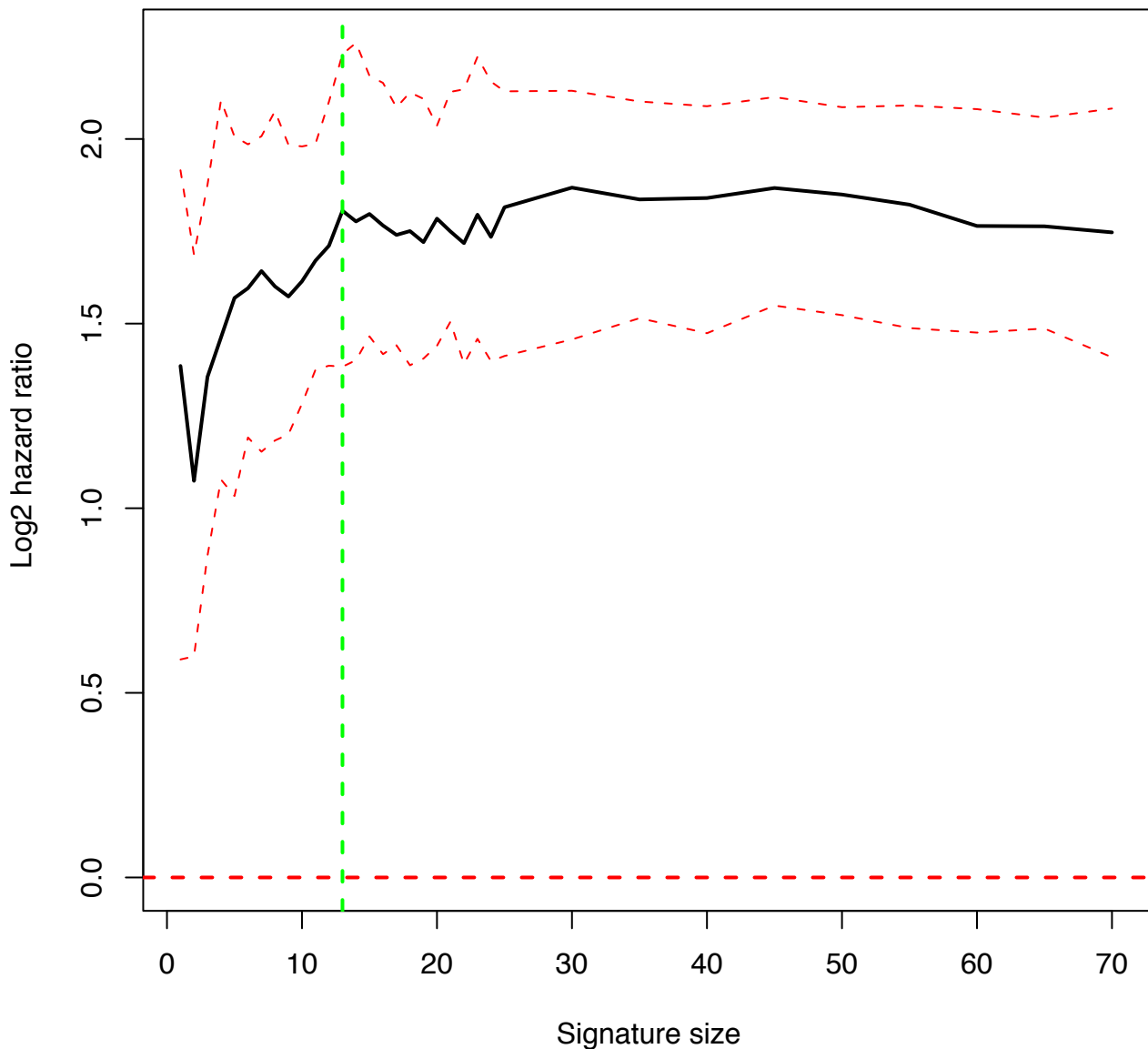

Supplement: Additional file 6 — External validation of the classifier (Ma, Reid and Jansen datasets). (a) Kaplan Meier curves for Ma et al. The risk group was defined by the classifier using a 50:50 cutoff. The two survival curves were not significantly different according to the logrank test (p-value of 0.1). (b) Kaplan Meier curves for Reid et al. The risk group was defined by the classifier using a 50:50 cutoff. The two survival curves were significantly different according to the logrank test (p-value of 0.05). (c) Kaplan Meier curves for Jansen et al. The risk group was defined by the classifier using a 50:50 cutoff. The two survival curves were significantly different according to the logrank test (p-value of 0.25). [file 1471-2164-9-239-S6.pdf]
